# Natural Appetite Control: Food-Derived Aromas as Appetite Decreasing Agents—A Proof-of-Concept Study

**DOI:** 10.3390/nu17050819

**Published:** 2025-02-27

**Authors:** Michaela Godyla-Jabłoński, Natalia Pachura, Marta Klemens, Julia Wolska, Jacek Łyczko

**Affiliations:** 1 Department of Human Nutrition, Wrocław University of Environmental and Life Sciences, ul. Chełmońskiego 37/41, 51-630 Wrocław, Poland; 2 Department of Food Chemistry and Biocatalysis, Wrocław University of Environmental and Life Sciences, ul. Chełmońskiego 37/41, 51-630 Wrocław, Poland; natalia.pachura@upwr.edu.pl (N.P.); marta.klemens@upwr.edu.pl (M.K.); 122508@student.upwr.edu.pl (J.W.)

**Keywords:** appetite regulation, sensory stimuli, overweight and obese, aroma, GC-MS

## Abstract

**Background and Objective:** The global population is struggling with significant health challenges, among which overweight and obesity stand out. Currently, 61% of adults and 7.5% of children and adolescents are affected, underscoring the urgent need for effective solutions. This study evaluated appetite-reducing prototypes related with food products, focusing on their ability to influence appetite through the sense of smell. The objective was to determine the effectiveness of these prototypes and identify the most promising candidates for further research. **Methods:** A questionnaire-based consumer survey was performed for six appetite-reducing agents. Forty-five participants with elevated body mass index values (BMI ≥ 25) were asked to verify the samples in terms of aroma intensity, pleasure, and potential for appetite reduction. Also, qualitative parameters such as the identification of the samples’ food associations was performed within the questionnaire. The questionnaire results were further compared with headspace solid-phase microextraction (HS-SPME Arrow) analysis results to identify volatile organic compounds associated with appetite-reducing properties. **Results:** The proof-of-concept study revealed that prototypes with unpleasant and irritating aromas demonstrated the highest appetite-reducing potential, scoring approximately 24 out of 35 points. Conversely, prototypes with pleasant, dessert-like aromas showed lower effectiveness, scoring between 14 and 18 points. **Conclusions:** By linking consumer perceptions to chemical analyses, we identified effective prototypes for further investigation, including studies measuring actual food intake. These findings contribute to developing innovative, non-invasive strategies to address overweight and obesity, offering a new dimension to appetite control through sensory modulation.

## 1. Introduction

Considering proper and healthy nutrition, appetite management is one of the major modern challenges for emerging and developed countries. Improvements in the fields of medicine and pharmaceuticals, food production, food safety, or overall life comfort, de-spite the unquestionable benefits, may bring some risks. One of them is a disturbed hunger–appetite relationship, which may result from an overly high appetite [1]. This observation was used by Lowe and Butryn [2] to create the term ‘hedonic hunger’—the hunger feeling based not on the physiological needs of the organism but on psychological aspects, often linked to cravings or eating disorders. The issue of high appetite is associated with the obesity and overweight pandemic. According to the World Health Organisation, in 2022, 43% of adults were overweight and 18% of adults were obese. Furthermore, almost 0.6 billion children and adolescents are also dealing with high body mass, which predicts that the problem will increase in the future.

To prevent the issues of overeating, a complex and comprehensive strategy should be used. Consumers should use appropriate physical activity and a healthy and balanced diet [3]. The constitution of the diet, despite the presence of proper and high-quality food, is also linked to adequate per-individual calorie intake. Unfortunately, not all consumers are able to control their calorie intake based on their daily routine, and thus, they require support [4]. At this moment, the market offers some solutions for people with both high and low appetites. Despite the presence of dietary supplements, whose efficiency is often doubtful, there are available pharmaceutical solutions, such as Saxenda^®^ (an analogue of the GLP-1 hormone) and Mysimba^®^ (a combination of naltrextone and bupropion) for overweight consumers and those with obesity who want to reduce their calorie intake [5,6]. However, these solutions have numerous limitations including price, availability regarding certain countries’ legal barriers, and side effects after usage [7,8,9,10].

One of the factors with crucial influence on calorie intake is the activity of AGRP (agouti-related protein) neurones, which, when disturbed, may influence high consumption. There have been scientific reports [11,12] that have pointed out that sensory stimuli may affect AGRP neurone activity, leading to appetite stimulation or appetite reduction. Such practical attempts in this matter based on olfactory stimuli were studied by Morquecho-Campos et al. [13,14]; however, the obtained results did not clearly answer the issue. Within their studies, Morquecho-Campos et al.’s team worked on the food-related aromas, namely those linked to high carbohydrate contents (corn and bread), high protein contents (duck and chicken), high fat contents (diacetyl for butter and cream), and low-energy products (cucumber and melon) and their influence on appetite and food intake. As they hypothesized, the specific smell related to a macronutrient group would increase the appetite for congruent foods. The studies revealed that active smelling may have influenced appetite; however, it did not affect actual food intake, and unconscious smelling did not have any effect. In another study cycle, Ogawa et al. [15,16,17,18], using animal models, investigated how the influence of the food-related volatile organic compounds (VOCs), which may be found in nutmeg, curry, vanilla, or cinnamon, due to specific structures, may increase appetite. Within those studies, it was proven that the presence of the phenylopropanoid group, the vanillin group, and side aliphatic chains in the structures influences the efficiency and time of effective influence on animal-model appetite. Finally, Abeywickrema et al. (2022) [19] have verified that the exposure of foods with strong vanilla aromas during breakfast may reduce the intake of sweet snacks or promote the intake of less-sweet snacks during the day.

Based on one of our earlier research studies [20], in which we investigated what types of foods aroma may influence the consumer’s level of appetite, we have composed prototypes of appetite-reducing agents (ARAs). On the broad survey addressed to overweight consumers and those with obesity (BMI ≥ 25), there were found over 60 food products and meals whose aromas were considered as having appetite-regulating potential. Based on HS-SPME-GC-MS emission analysis, we have established a group of VOCs that consisted of various food products and meals. Identified VOCs were linked to fruity aromas (e.g., esters), floral notes (e.g., linalool), herbal notes (e.g., pinenes, camphor, and carvone), balsamic notes (e.g., 2-acetylfuran), or aldehydic, fatty notes (e.g., linear aldehydes and linear alcohols). Those findings helped us build a hypothesis that aromas connected with food products and meals that may be associated with safety, bliss feelings, and satiety would have potential to reduce appetite while those aromas would not be repelling, disgusting, and irritating. In this paper, we would like to propose a proof-of-concept study for seek of appetite control agents that would have an influence on appetite reduction. For this purpose, six ARAs with different compositions and characteristics were subjected to questionnaire study, during which 45 people with elevated BMI values (≥25) evaluated the ARA samples in terms of aroma quality and potential to reduce appetite.

## 2. Materials and Methods

### 2.1. Chemicals and Reagents

All chemicals and reagents used for this study were purchased at Sigma-Aldrich (Steinheim, Germany). The food flavoring agents used as a base for parts of ARAs were obtained from Super Aromas, Sobucky Poland sp. Z o.o. Sobucky Ltd., Sp.k. (Lublin, Poland), smoke aroma from OLMIX s.c., (Kozy, Poland), and essential oils from Herbiness Inez Rogozińska (Chomiec, Poland).

### 2.2. Appetite-Regulating Agents for Appetite Reduction (ARAs)

Six ARAs with potential for appetite reduction were used for this study. The selection of used ARAs was based on our previous study [20]. Briefly, in the mentioned study, twenty-five ARAs prototypes were composed and evaluated by trained sensory panel in terms of aroma quality, intensity, and the potential to manage appetite. On the base of the panel evaluation, six ARAs were qualified as ones for further studies. The ARA codes and composition are given in Table 1. The different amounts of used samples, S005 and R789, were dictated by the sample odor intensity, which was very low if 40 µL was used. The volatile profiles of investigated ARAs are given in Supplementary Materials’ HS-SPME-GC-MS results (Table S1 X034, Table S2 H090, Table S3 F699, Table S4, S005, Table S5 R789, and Table S6 D418).

### 2.3. Participants

All ARA samples were stored in dark 100 mL glass bottles with plastic caps. Immediately prior to consumer testing, the amounts of ARA samples specified in Table 1 were applied to blotter paper (2 × 0.5 cm) and placed in odorless 2 mL Eppendorf-type tubes. Each tube was signed with the specific ARA sample code and delivered to consumers for testing.

The research was carried out in accordance with the Code of Ethics of the World Medical Association (Declaration of Helsinki). Ethical approval for the involvement of human subjects for this study was granted by Wrocław Medical University Research Ethics Committee, reference number KB-677/2020, 26.11.2020r.

A group of consumers consisting of 45 individuals were invited to participate in the proof-of the concept study. The possibility to participate in the study was announced by social media and via the internal university newsletter (for university employees and students). The study was conducted in the first quarter of 2024 and the following were set as requirements for taking part in the study: increased BMI (≥25); not being on a vegan, vegetarian, or other diet that excludes large food groups; having lived in the Central European region for at least 5 years; and having no known anosmia, hypernosmia, or other serious olfactory disorders. Within this study, only individuals with increased BMI (≥25) were considered, and no control group with regular BMI (18.5–24.99) was considered since we were interested in the influence of ARAs on the appetite of overweight people and those with obesity, and thus, regular BMI control group would not have brought any significant insights on this matter. Such approach was earlier used in Proserpio et al.’s [21] study.

The qualification of participants for the study was carried out by a committee acting within the framework of the implementation of the project of which the presented study was a part. The consumer group size was calculated based on previous studies focused on evaluation of aroma influence on appetite [13,14,22], supported with statistical analysis of sample size with assumed root of the sum of squares of standardized effects as 0.25, test power as 0,8 and alpha as 0.05. The statistical prediction is given in Supplementary Materials section on statistical assumptions.

The characteristics of the study group was as follows. Thirty-six women and nine men aged 19–58 years participated in the study, among whom 19 were overweight (BMI: 25.00–29.99 kg/m^2^) and 26 had obesity (BMI ≥ 30.00 kg/m^2^). Most respondents lived in cities with more than 250,000 inhabitants (*n* = 28), 9 people resided in cities with up to 100,000 inhabitants, and 8 participants lived in villages. The highest proportion of people were in higher education (*n* = 34, 75.6%), 9 participants (20.0%) had vocational education, and only 2 people (4.4%) had primary education. Most respondents had eaten their last meal between 1 and 3 h before the start of the study (*n* = 28). Only 15 people had eaten their last meal more than three hours before the start of the survey, and only two participants had been eating something less than an hour before the survey.

### 2.4. Questionnaires and Consumer Study Procedure

The questionnaires were given to the participants in their native language, Polish. Translated to English questionnaire, this has been provided in Supplementary Materials: Questionnaire section.

Before the study, participants were acquainted with written informed consent and the instructions. Consumers conducted sensory evaluation in individual booths, in which were controlled illumination (70–90 footcandles) and temperature (23 ± 2 °C) [23]. Besides conditions, each chute was equipped with instructions (placed on the wall in front of the seat; the transcript of the instructions is given in Supplementary Materials: Instructions for Participants). Every participant was given a set containing 7 samples, including one being the control. Complete set for each participant included a set of samples, a dedicated questionnaire, and one tea bag (Lipton Yellow Label, PepsiCo, Harrison, NY, USA). Due to the similarity of one sample to coffee, we used tea as a “nasal palate cleanser” for reducing the olfactory adaptation and habituation effects. The participants were instructed to open each sample and sniff it and, thereafter, to keep constantly sniffing from the Eppendorf tube to fill up the questionnaire. Between sniffing each sample, cleaning their noses by sniffing tea bags was suggested. Time taken for the consumer evaluations fluctuated from 45 min to 1 h and 15 min; overall, participants completed the test in 1 h.

During consumer studies, 45 repetitions for each sample were collected.

Each questionnaire involved metrics, with 7 sheets of forms (one form per sample), every one of them marked with a code number. Questions (Q1–Q6) 1–2 asked about liking the sample, including its intensity, asking for answers on a scale from 1 to 5, which is a well-known scale for consumers in Poland. Questions 3–5 focused on an aspect related to reducing the appetite; if answered positively, the follow up question asked about three verbs (food-related) associated with the smell. Last (sixth) question asked about three adjectives in relation with the smell of the sample. Last sheet was an independent form—the ranking test, to conclude all the samples. There were two tables. In the first one, consumer had to align samples due to their liking of the sample, from least liking to most, and the second focused on reducing effects, same as the previous one, from least to most. The questionnaire was pre-tested by the group of 10 randomly invited individuals from among academic stuff and students. Based on their suggestions, some questions were clarified, and visual aspects were corrected before the actual study. Based on pre-test answers, the internal consistency of the questionnaire was evaluated by application of Cronbach’s alpha coefficient, which was 0.80, and thus, it was evaluated as acceptable to proceed with the study. The data for Cronbach’s alpha coefficient calculation are given in Supplementary Materials section on statistical assumptions.

Questions:

Q1: How do you generally like the scent of this sample? (scale: 1–5 points)
Q2: How do you rate the intensity of the scent of this sample? (Choose “Bad”, “Hard to say”, or “Good”; if you have chosen “Bad” or “Hard to say”; we request you to respond to Q2*.)
Q2*: The scent is: (scale: 0–5 points; 0 if answer for Q2 was “Good”).
Q3: Does the presented scent remind you of an appetizing meal and/or food? (Scale: 1–5; if one scored 4 or 5; we request you to answer Q3*.)
Q3*: What meal or food does the scent of this sample remind you of? Please list up to 3 items.
Q4: Does the scent of this sample evoke a sense of contentment and/or state of being secure for you? (Choose “No”, “Hard to say”, or “Yes”.)
Q5: Please indicate to what extent the scent of this sample could reduce your appetite (scale 1–5).
Q6: Please describe the scent of this sample with three adjectives that best resonate with you.

### 2.5. Questionnaire Data Interpretation

#### 2.5.1. Questions with Numerical Scale

All questions where the respondents had to evaluate some measurable features such as odor intensity or the level of pleasure were assigned with a 1–5-point scale with additional explanation regarding what scores meant (Q1, Q2*, Q3, and Q5). For questions with the need to choose “Bad”, “Hard to say”, and “Good” or “No”, “Hard to say”, and “Yes” possibilities (Q2 and Q4, respectively), the answers were translated to −5, 0, or 5 points. Finally, if the Q2 question answer was “Good”, the points in Q2* would equal 0.

#### 2.5.2. Descriptive Questions

Descriptive questions where respondents were asked about their associations with ARAs were treated qualitatively, and for data interpretation, the commonly used descriptions were used.

#### 2.5.3. Compare and Contrast Assignment

During the last task, the respondents were asked to rank the samples according to increasing odor pleasure or potential for appetite reduction. Since the consumers had to evaluate 7 samples, during the data interpretation, the positions were assigned with points from 5 up to 35, with each step increasing by 5 points. In the rare cases when the respondents had placed multiple samples on some rank (leaving some ranks without a response), all samples placed on rank received points assigned to this rank.

### 2.6. HS-SPME-GC-MS

To screen for the chemical composition of ARAs, GC-MS analysis was performed by headspace solid-phase microextraction Arrow (HS-SPME Arrow) based on [20]. The composition of individual prototypes is available in Supplementary Materials section on HS-SPME-GC-MS results. A blotter covered with the appropriate amount of prototype was placed in a 20 mL headspace vial along with an internal standard (2-undecanone, Sigma-Aldrich, Germany). The sample was then incubated for 5 min, at 30 °C, to be followed by extraction for 10 min using 1.10 mm DVB/C-WR/PDMS SPME Arrow fiber (Shimadzu, Kyoto, Japan). Subsequently, the analytes were desorbed for 3 min at 250 °C with a split of 150. Separation of individual analytes was carried out on a ZB-5MSi (30 m × 0.25 mm × 0.25 μm) column (Phenomenex, Torrance, CA, USA). The operating conditions of the GC-MS were as follows: helium as carrier gas with flow 1 mL min^−1^; temperature program: 50 °C as initial temperature, then 130 °C at a rate of 4.0 °C min^−1^, then 180 °C at a rate of 10.0 °C min^−1^, then 280 °C at a rate of 20.0 °C min^−1^; interface temperature: 250 °C; ion source temperature: 220 °C, and scanning mode: 40 to 400 *m/z*.

Identification of volatile compounds found in the ARAs was carried out by comparing both the experimentally obtained linear retention indices (LRIs), which were calculated using the C_7_-C_40_ *n*-alkane mix (Sigma-Aldrich, Germany), as well as the obtained mass spectra with those available in the GCMS Library of Natural and Synthetic Compounds of Flavour and Fragrance (FFNSC 3) and the NIST 23 Mass Spectral and Retention Index Libraries (NIST23) (National Institute of Standards and Technology, Gaithersburg, MD, USA). Only those with mass spectral similarity ≥ 90% and LRI ± 15 were considered as potential compounds.

Instrumental analyses (HS-SPME-GC/MS) were performed in triplicates.

### 2.7. Statistical Analysis

For data analysis, Statistica 13.3 (StatSoft, Kraków. Poland) software was used. The data obtained during consumers studies were analyzed by Tukey’s Honest Significant Difference test (Tukey’s HSD) and by multivariate analyses—hierarchical cluster analysis (HCA) and grouping for features and for objects (heatmap). For HCA analysis, the analytical procedure was performed by applying Ward’s method and Euclidean distance, while the interpretation was carried out with strict (33%) Sneath’s criterion. The Sneath criterion has been used in a formula-based approach, making it a quantitative method of identifying clusters instead of relying solely on visual inspection, allowing for greater control and consistency in HCA, enabling more reliable identification of clusters in complex datasets. For heatmap visualization, the crude data were standardized according to software algorithms. For verification of interference of other than samples’ aroma factors, analysis of variance (ANOVA) for factors such as sex, age, BMI, time of last meal, and feel of hunger and ANOVA for system of factors was conducted. Moreover, the Pearson correlation coefficient was calculated for pleasure of aroma, potential to reduce appetite, and anthropometric factor expressed as BMI.

## 3. Results

Within the questionnaire study, 45 consumers had evaluated six ARAs and a control sample. The questionnaire questions were focused on the aromas’ descriptions, aroma pleasure assessment, and the potential to reduce appetite. Table 2 gives a brief overview of the descriptive part of the questionnaire. Overall, the consumers had no trouble linking the particular ARAs with food examples and describing their aromas. By the qualitative description, the samples D418 and R789 were assessed as unpleasant ones, with artificial, chemical characteristics. On the other hand, F699, H090, S005, and X034 were determined as samples with pleasant aromas, which were linked with satisfying, fruity, or refreshing foods. The results of L368, the odorless control sample, showed that the consumer group was able to perform the test.

**Figure 1:**
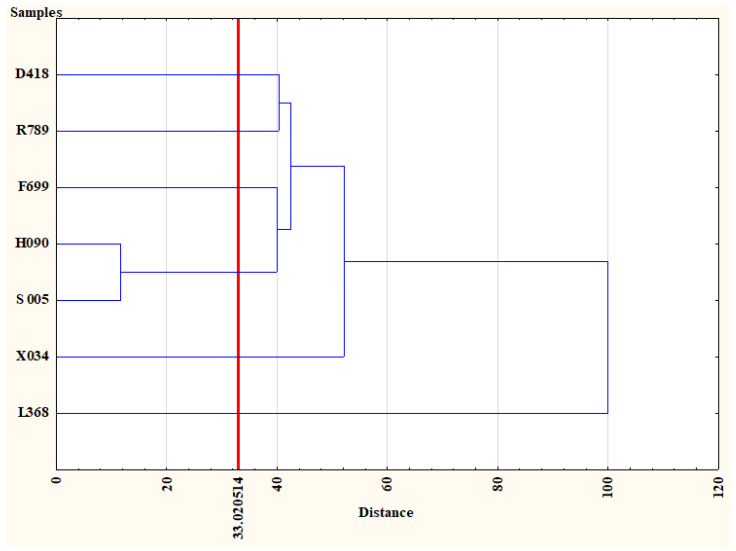
HCA results of sample classification based on the scoring questions (Q1: “How do you generally like the scent of this sample?”, Q2: “How do you rate the intensity of the scent of this sample?”, Q2*: “The scent is?”, Q3: “Does the presented scent remind you of an appetizing meal and/or food?”, Q4: “Does the scent of this sample evoke a sense of contentment and/or state of being secure for you?”, and Q5: “Please indicate to what extent the scent of this sample could reduce your appetite”). Applied for sample classification, Sneath’s criterion (33%) has been marked with red vertical line. x-axis represents the similarity index of the samples and x-axis represents the samples. The HCA classification showed that the appetite-reducing agents, due to the consumer evaluation, could be divided for six groups, where only H090 and S005 samples were classified commonly. The control sample (L368) presented a fully separate group. Data were based on responses of 45 consumers with BMI ≥ 25. HCA: hierarchical cluster analysis; BMI: body mass index.

**Figure 2:**
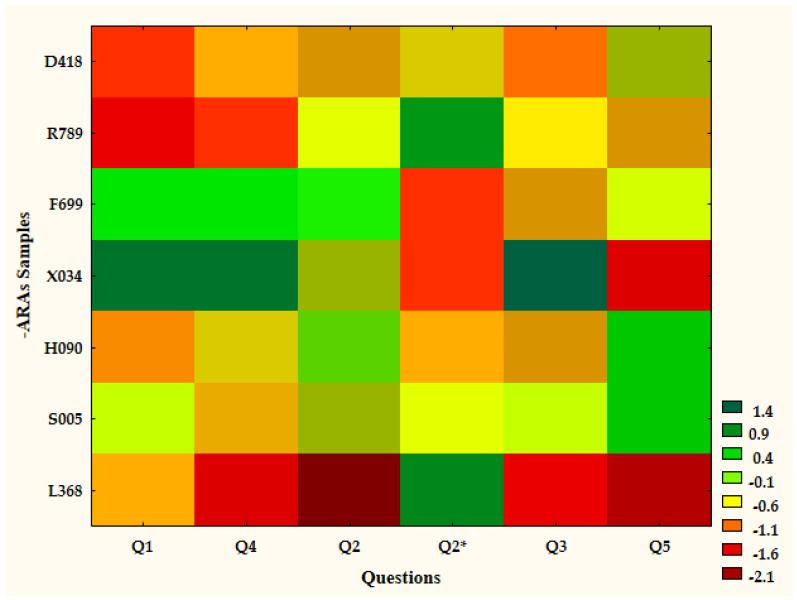
Heatmap illustrating the distribution of points awarded by consumers (45 individuals with BMI ≥ 25). The heatmap was created based on standardized (through the statistical software algorithm) data to reduce the impacts of the different scoring scales used for questions. Therefore, despite the differences in scoring scales, the applied color spectrum used is consistent. In the color legend provided, the green shades represent the lowest scores, the yellow shades the moderate scores, and the red shades the highest scores. The green shades are related to preferable parameters. The raw numbers and standardization matrix can be found in Supplementary Materials section on statistical assumptions, Table S8. x-axis and y-axis represent the plotted variables; x-axis shows questions; y-axis shows samples. The heatmap highlights the similarities between F699 and X034 samples, the similarities between samples D418 and R789, and the similarities between samples H090 and S005. The control sample (L368) shows the separate characteristics.

**Figure 3:**
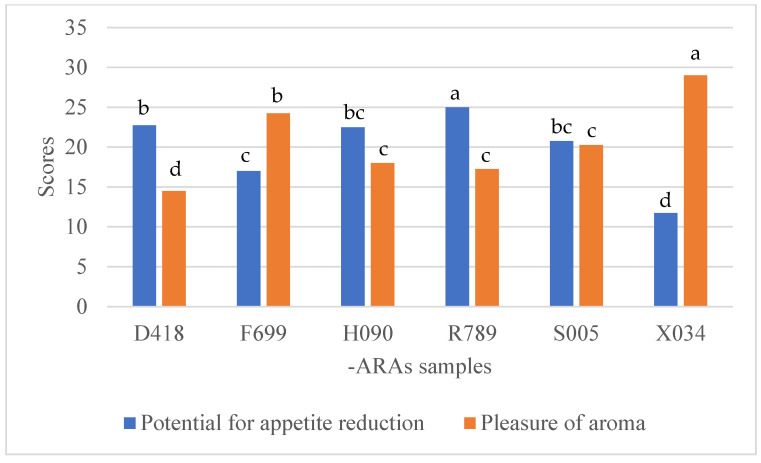
Evaluation of appetite-reducing agents by consumers with obesity (26 individuals with BMI > 30) in terms of sample pleasurable aroma and appetite reduction. Values followed by the same letters (a, b, c, or d) were not statistically different in Tukey’s HSD within the data series. Samples D418 and R789 received significantly lower scoring than other samples in terms of the aroma pleasure; however, that resulted in significantly higher potential to reduce appetite. Sample X034 showed fully reversed tendency. Samples F699, H090, and S005 demonstrated moderate results for pleasure of aroma and potential to reduce appetite.

The results of HCA showed that the questionnaire descriptive questions had their reflection in the scoring questions. According to Figure 1, the L386 sample (control) was fully separately clustered from ale samples with actual aromas. In the second linking point, the X034 sample, assessed as undoubtfully the most pleasant one was separated from the others. Further, samples D418 and R789 were separated due to their unpleasant and irritating aromas. Finally, the last two clusters were made of F699 and H090 paired with S005. The heatmap in Figure 2 shows, in detail, the distribution of points granted by the consumers. Q1 and Q4 referred to the overall pleasure of the sample aroma and the association with safety and blissful feelings, respectively. Clearly, the L368 (control), D418, and R789 samples received the lowest scoring, followed by the H090 and S005 (average scoring) samples, while F699 and X034 received the highest points. Also, similar differences were observed for Q3, where we asked if the sample aroma may have been associated with a satiating food or meal. Finally, in terms of appetite-reducing potential (Q5), the situation changed and the samples F699 and X034 presented the lowest potential (near to that of the control sample) while D418, R789, H090, and S005 earned higher points. Moreover, the Figure 3 shows that samples H090 and S005 present, simultaneously, similarlevels of aroma pleasure and appetite reduction potential.

**Figure 4:**
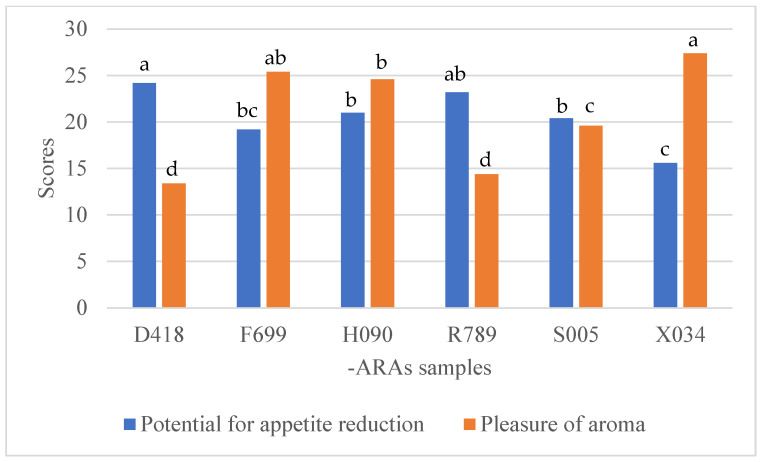
Evaluation of appetite-reducing agents by overweight consumers (19 individuals with 25 ≤ BMI ≥ 30) in terms of sample pleasurable aroma and appetite reduction. Values followed by the same letters (a, b, c, or d) were not statistically different in Tukey’s HSD within the data series. Samples D418 and R789 received significantly lower scoring than other samples in terms of the aroma pleasure; however, that resulted in significantly higher potential to reduce appetite. Sample X034 showed fully reversed tendency. Samples F699, H090, and S005 demonstrated moderate results for pleasure of aroma and potential to reduce appetite.

The most crucial was the evaluation of the levels of the samples’ pleasurable aromas considering their appetite-reducing potential. The scheme in Figure 3 and Figure 4 show the relationship between the levels of samples’ pleasurable aromas and their appetite-reducing potential considering the level of BMI excess. Overall, overweight consumers and consumers with obesity showed similar tendencies during ARA sample evaluation. According to our results, the samples with the highest notes in terms of aroma pleasure, at the same time, presented lower potential to reduce appetite (F699 and X034) while the samples with odd and irritating aromas (D418 and R789) showed the highest potential to reduce appetite. The middle ground was given to the H090 and S005 samples, which demonstrated similarities between the levels of aroma pleasure and appetite-reducing potential.

## 4. Discussion

The evaluation of aroma potential to moderate the human appetite is a complex issue due to the multiple factors at play such as individual odor perceptions, odor associations, food cultures, and others. Nonetheless, some trends corresponding to previous research were observed. First, the unpleasant and irritating ARAs (R789 and D418), which, according to Table 2, may have been associated with less appetizing foods (oyster sauce or soy sauce), were considered by consumers to be those with the highest potential to reduce appetite. This agrees with Piqueras-Fiszman et al.’s [24] results on negative and non-negative food pictures’ influence on appetite and, moreover, this was considered by Boesveldt and de Graaf [25] in their review. On the contrary, the samples with pleasant odors, such as X034 and F699, presented lower potential to reduce appetite. However, sample F699 was also described as ‘irritating’ by consumers; however, the overall description was different from those of R789 and D418. The F699 sample showed a more fruity character without nonpopular notes such as of rubber or oyster sauce. The irritating effect may have been related to minty aromas, which, with longer exposure, may become less acceptable.

The reason for that may have been found in their odor character. Zoon et al. [22] have established that odors may stimulate appetite for particular or similar foods. Therefore, we think that since consumers within this study did not actually eat food but only in theory evaluated the potential of ARAs. The associations of pleasant, chocolate-like, and dessert-like (more in Table 2) aromas for X034 was linked with sweets that do not coalesce with appetite reduction. Furthermore, in a series of studies, Ogawa and Ito [15,16,17,18] have shown that compounds with particular structures in their molecules, such as the vanillin group or phenylpropanoids, have significantly greater potential to stimulate appetite than other compounds. The compounds investigated by Ogawa and Ito’s [19,20,21,22] team may be found in vanilla, chocolate, nutmeg, or curry, and thus, the chocolate-like and dessert-like linked aroma of the X034 sample may have been related to that issue. On the other hand, Abeywickrema et al. [19] proved, in their study with consumers, that exposure to a high-intensity vanilla aroma during breakfast led to less sweet-snack consumption during the day; however, the amount of snack intake did not change. Therefore, our hypothesis that the aroma of foods with high calorie concentrations will reduce appetite, since the organism will prepare for high doses of energy, stays, at this moment, unsolved since it is not clear what the actual effect of X034 use would be when actual food intake were measured.

In the case of the F699 sample, the consumer associations were linked to fruits, smoothies, and a refreshing experience, which may have been linked with juiciness, which, overall, also may stimulate appetite rather than reduce it. On the other hand, the main compound in the volatile profile of the sample F699 (Supplementary Materials: HS-SPME-GC-MS results, Table S3) was carvone, which, in studies using animal models, was suspected to cause appetite reduction [26]. This contradiction may be just apparent due to the fact that consumers participate in studies with their individual mindsets, which may be created by various non-controlled (by the experimental framework) factors such as mood, recently consumed meals, and personal beliefs and routines. Nonetheless, according to Morrin and Tepper’s (2021) [27] studies, at present, one needs verify what is the difference within the assumptions made in fully controlled experiments and consumer studies. This perfectly shows the need for proof-of-concept studies, which allow us to verify basic hypotheses, especially when experiments are dedicated to being basic points for further, large-scale studies.

The most interesting, in terms of appetite reduction, were samples S005 and H090. Both presented moderate potential to reduce appetite and moderate evaluations of aroma pleasure. The sample S005 presented a strongly minty odor character, what may have been linked again with the presence of VOCs such as carvone, menthol, or menthone, which, in earlier studies on animals, were suspected of appetite-reducing properties [26,28]. Furthermore, the research of Miyazaki and Kawahara [29] has shown that mint-scented masks have a potential to reduce appetite. Meanwhile, the H090 sample was more like the F699 sample; however, it was less spicy, intensive, and lemon-like (H090 was overall described as citrus-like), which overall led it to provide better results in terms of appetite reduction.

This result corresponded with results presented the broad review prepared by Nguyen et al. [30]. As the authors of the review showed systematically, the presence of particular compounds in essential oils resulted in appetite increasing or decreasing. Namely, the presences of limonene, eucalyptol, citral, and beta-citronellol were linked with decreased appetite. Moreover, Nguyen et al. [30] pointed out that citrus essential oils or mint essential oils had positive effects on appetite reduction. With reference to the present study, such compounds, or aromas represented by them, may be found in the S005, H090, and F699 ARAs. Sample S005 was characterized with 23.24% of limonene, 17.09% of menthone, and 6.68% of isomenthone; sample H090 was characterized with 50.46% of limonene and sample F699 was characterized with 21.94% of carvone and 7.49% of citral. Also, those compounds were responsible for aroma notes that could be found in fragrances analyzed by Miyazaki and Kawahara [29] and Nguyen et al.’s [30] studies, namely mint-like notes and citrus-like notes.

In addition, studies and reviews on gene expression [31] and olfactory receptor variation [32] have shown that compounds such as limonene or linalool can affect appetite. Shen et al. (2005) [33], using an animal model, showed that grapefruit essential oil, the main component of which is limonene, can affect the activity of nerves associated with the stomach, which can delay the removal of food, which then leads to a longer feeling of satiety. Interestingly, Shen et al. (2005) [33] verified that reducing nasal receptor activity reduced the level of effect on nerves. Another branch can be linked to Chen et al. (2012) [31], who proved the effect of limonene on neuropeptide Y mRNA expression, which was suppressed by the volatile compound. This pathway clearly explains samples S005 and H090, whose volatile profiles contained about 23% and 50% limonene, respectively.

The limitation of the present study was the theoretical, questionnaire-based level of ARA efficiency evaluation. Nonetheless, such issues, according to Girona-Ruíz et al. [34], may be commonly found within scientific studies. Moreover, a detailed ANOVA analysis (Supplementary Materials: ANOVA, Tables S9 and S10) showed that other factors besides the sample flavor, such as the consumer’s gender, age, BMI, time since last meal, or feeling of hunger, had no significant effects on sample ranking. This lack of significant differences was also observed after conducting an ANOVA for the factor system. These results may be surprising, especially since gender as a differentiating factor is often used to verify flavors. Nevertheless, this issue is more often observed not when describing aroma qualitatively, but when assessing aroma intensity, as demonstrated by Rosa et al. [35] and Sollai et al. [36]. In the case of aroma enjoyment and effects on appetite, cultural factors may be more significant. Additionally, it seems that despite the level of BMI (overweight or obesity), consumers similarly evaluated the aromas of ARAs and the potential for appetite reduction by them. However, participants with BMI values above 30 (obesity) showed less differentiation between the samples, which may suggest that more excessive weight lowers sensitivity to the smells of ARAs. Finally, due to organizational constrains, it is necessary to preform proof-of-concept studies, which will allow us to reduce the numbers of samples for further studies, which may be conducted under real-life conditions.

## 5. Conclusions

The present proof-of-concept study verifies the appetite control agents in terms of lowering appetite potential. In this consumer study, participants were given six compositions and an unscented control sample, which were evaluated by consumers based on a theoretical questionnaire. We assumed that samples with pleasant, dessert-like aromas would have the highest appetite-reducing potential due to their effect on the pleasure center in the brain and fooling the human body into thinking that the need for food intake had already been satisfied. Meanwhile, samples with irritating and generally unpleasant aromas (R789 and D418) had the highest potential for appetite reduction, being associated with unpleasant sensations and rather unsatisfying foods such as oyster sauce and soy sauce. In contrast, samples with pleasant desert-like (X034) or candy-like (F699) aromas were the least appetite-reducing according to the consumers. In the middle, with moderately pleasant aromas and moderate appetite-reducing potential, were samples with minty, citrusy aromas (S005 and H090). One of the main compounds in the volatile profiles of S005 and H090 was limonene, which had been previously identified as a compound that, through olfactory receptors, affects the gastric nervous system and the expression of neuropeptide Y-related mRNA, leading to an effective reduction in appetite.

For further evaluation, the samples with balanced relationships between aroma pleasure and potential for appetite reduction, namely F699, S005, and H090, will be subjected to advanced-stage studies, during which actual food intake after exposure to consumers for aroma investigation and the optimal conditions of exposure will be investigated.

## 6. Patents

Michaela Godyla-Jabłoński, Natalia Pachura, Marta Klemens, and Jacek Łyczko have patent applications P.445247, P.445248, and P.445252, pending at the Wrocław University of Environmental and Life Sciences.

## Figures and Tables

**Table 1:** Sample codes, sample composition, and sample quantity used for consumer study.

Sample Code	Components	Volume of Particular Components Per 100 mL of ARAs * [mL]	Amount of Sample Used for Consumer Study [µL]	Number of Identified with HS-SPME-GC-MS Volatiles (Supplementary Materials: HS-SPME-GC-MS Results)
D418	nonanoic acid	0.5	40	11
	6-methyl-5-hepten-2-one	0.5		
	2-heptanon	0.5		
	decanal	0.5		
	rosemary essential oil	0.15		
	triethyl citrate	97.85		
X034	dessert coffee aroma	10	40	14
	2,5-dimethylpyrazine diluted 100×	0.1		
	2,6-dimethylpyrazine diluted 100×	0.1		
	2-ethyl-3-methylpyrazine diluted 100×	0.1		
	chocolate aroma 20 mL	20		
	triethyl citrate	69.7		
H090	pineapple aroma	5	40	21
	limonene	2.5		
	myrcene	1.5		
	6-methyl-5-hepten-2-one	0.5		
	linalool	0.5		
	triethyl citrate	90		
S005	2,5-dimethylpyrazine diluted 100×	0.1	60	38
	2,6-dimethylpyrazine diluted 100×	0.1		
	2-ethyl-3-methylpyrazine diluted 100×	0.1		
	mint aroma	10		
	triethyl citrate	89.7		
R789	smoke aroma	5	60	12
	dodecanal	1		
	α-pinene	1		
	furfural	1		
	triethyl citrate	92		
F699	lemongrass essential oil	2.5	40	35
	6-methyl-5-hepten-2-one	0.5		
	carvone	2		
	α-phellandrene	0.5		
	verbenone	0.5		
	triethyl citrate	94		
L386 (control)	triethyl citrate	100	40	-

* ARAs—appetite-reducing agents; HS-SPME-GC-MS: headspace solid-phase microextraction coupled with gas chromatography and mass spectrometry.

**Table 2:** The summary of questionnaire descriptive questions (Q3*: “What meal or food does the scent of this sample remind you of? Please list up to 3 items” and Q6: “Please describe the scent of this sample with three adjectives that best resonate with you”) for specific ARA samples based on the responses of 45 individuals with BMI ≥ 25.

Sample	Food Association	Odor Description
D418	refreshing beverage; chewing gum; dessert; geranium; hard candies, sweets, lemon smoothie; oyster sauce; toilet cube	chemical; intensive; irritating; artificial; citrus-like;
F699	cocktail; yoghurt; sour jelly; green tea; lemon; hard candies; sorbet; smoothie; grapefruit; lemonade; mint;	intensive; refreshing; pleasant; irritating; lemon-like; spicy;
H090	ice-cream; fruit jelly; citrus juice; fruit dessert; citrus tea; fruit cake; tropical fruits; IPA beer; cherries;	citrus-like; fresh; intensive; refreshing; fruity; pleasant; sweet;
L368 (control sample)	no associations	neutral; bland; weak;
R789	almond biscuits; rubber; bland; soy sauce; fish; broth;	chemical; unpleasant; artificial; pungent; pharmaceutical; marzipan-like;
S005	mint chocolate (“5 o’clock”), ice-cream; apple; hard candies; chewing gum; mouthwash	minty; intensive; cooling; refreshing; herbal; fresh;
X034	vanilla; chocolate biscuit; cappuccino; tiramisu; coffee; dessert; cocoa; custard pie;	intensive; sweet; blissful; pleasant; chocolate-like;

## Data Availability

The raw data obtained from the consumer survey is available on the Open Science Framework website at DOI 10.17605/OSF.IO/KPW8R.

## Supplementary Materials

The following supporting information can be downloaded at: https://www.mdpi.com/article/10.3390/nu17050819/s1, Supplementary Materials: Statistical Assumptions; Supplementary Materials: Questionnaire; Supplementary Materials: Instructions for Participants; Supplementary Materials: ANOVA; and Supplementary Materials: HS-SPME-GC-MS Results.

## Abbreviations

The following abbreviations have been used in this manuscript:

ANOVA	Analysis of variance
ARAs	Appetite-reducing agents
BMI	Body mass index
HCA	Hierarchical cluster analysis
HS-SPME-GC-MS	Headspace solid-phase microextraction coupled with gas chromatography and mass spectrometry
LRIs	Linear retention indices
VOCs	Volatile organic compounds
