# Supplementary material for: Natural Appetite Control: Food-Derived Aromas as Appetite Decreasing Agents—A Proof-of-Concept Study
Source: Nutrients. 2025 Feb 27;17(5):819. doi: 10.3390/nu17050819 (PMC11901767; doi:10.3390/nu17050819)
Supplement: Supplementary file 1 [file nutrients-17-00819-s001.zip › nutrients-3488088-supplementary.pdf]

Supplementary materials: Questionnaire

Natural Appetite Control: Food-Derived Aromas as Appetite Decreasing Agents - a Proof-of-Concept Study

Michaela Godyla-Jabłoński [1], Natalia Pachura [2], Marta Klemens [2], Julia Wolska [2] and Jacek Łyczko [2,*]

[1] Department of Human Nutrition, Wrocław University of Environmental and Life Sciences, ul. Chełmońskiego 37/41, 51-630 Wrocław, Poland; michaela.godyla@upwr.edu.pl

[2] Department of Food Chemistry and Biocatalysis, Wrocław University of Environmental and Life Sciences, ul. Chełmońskiego 37/41, 51-630, Wrocław, Poland; natalia.pachura@upwr.edu.pl; marta.klemens@upwr.edu.pl; 122508@student.upwr.edu.pl; jacek.lyczko@upwr.edu.pl

*Corresponding author. Email: jacek.lyczko@upwr.edu.pl

LIDER PROJEKTU
Uniwersytet Przyrodniczy we Wrocławiu
Ul. C.K. Norwida 25
50-375 Wrocław

Dear Sir/Madam,

Thank you for your willingness to participate in our study. During today's study entitled "Consumer research on appetite-regulating agents in conditions close to real-life settings within the framework of the LIDER XI project titled "New generation appetite active agents – useful for long-term care and weight control" you will receive 6 coded scent samples to smell. The samples will be provided to you one after the other, in random order. You will have approximately 7-8 minutes to assess each sample.

We kindly ask you to smell each sample and then complete the assigned questionnaire (please match the code on the sample with the one in the upper right corner of the questionnaire sheet). Please provide your responses while being exposed to the scent and ensure that your answers are spontaneous.

At the very end, when you have all the samples in your booth, please fill out the questionnaire titled "Comparison."

If you have any doubts or questions, please feel free to contact the members of the research team conducting the study.
The results obtained in the study may be used for the preparation of a scientific publication and/or grant application and/or other related activities.

On behalf of the research team,
Principal Investigator
Jacek Łyczko, PhD

NCBR Narodowe Centrum Badań i Rozwoju

„Nowej generacji środki regulujące apetyt – użyteczne w opiece długoterminowej oraz kontroli masy ciała" Akronim RANG.e
Projekt finansowany przez Narodowe Centrum Badań i Rozwoju
Numer umowy: LIDER/48/0191/L-11/19/NCBR/2020

LIDER

SAMPLE CODE

1. **How do you generally like the scent of this sample?**

	1	2	3	4	5
	I don't like it at all	I don't like it	Neutral	I like it	I like it very much
Mark □					

2. **How do you rate the intensity of the scent of this sample?**

	Bad	Hard to say	Good
Mark □			

If you marked **"bad"** or **"hard to say"** please provide your response in the table below. If you marked "good" please proceed to question 3.

The scent is:

	1	2	3	4	5
	Definitely too weak	Too weak	Appropriate	Too intense	Definitely too intense
Mark □					

NCBR Narodowe Centrum Badań i Rozwoju

„Nowej generacji środki regulujące apetyt – użyteczne w opiece długoterminowej oraz kontroli masy ciała" Akronim RANG.e
Projekt finansowany przez Narodowe Centrum Badań i Rozwoju
Numer umowy: LIDER/48/0191/L-11/19/NCBR/2020

LIDER

3. **Does the presented scent remind you of an appetizing meal and/or food?**

	1	2	3	4	5
	Very weakly	Weakly	Hard to say	Strongly	Very strongly
Mark					

If you marked **"4 - strongly"** or **"5 - very strongly"** please answer the following question. If you marked "1 - very weakly" or "2 - weakly" or "3 - hard to say" please proceed to question 4.

What meal or food does the scent of this sample remind you of? Please list up to 3 items: ………………………………………………………………………………………………

4. **Does the scent of this sample evoke a sense of contentment and/or state of being secure for you:**

	No	Hard to say	Yes
Mark →			

5. Please indicate to what extent the scent of this sample could reduce your appetite

		☐ Mark
1	Definite lack of appetite reduction	
2	Rather lack of appetite reduction	
3	Hard to say	
4	Noticeable appetite reduction	
5	Strong appetite reduction	

6. Please describe the scent of this sample with three adjectives that best resonate with you:

NCBR Narodowe Centrum Badań i Rozwoju

„Nowej generacji środki regulujące apetyt – użyteczne w opiece długoterminowej oraz kontroli masy ciała" Akronim RANG.e
Projekt finansowany przez Narodowe Centrum Badań i Rozwoju
Numer umowy: LIDER/48/0191/L-11/19/NCBR/2020

LIDER

Personal Information:

1. Gender:

Female:		Other (specify):	
Male:		I prefer not to specify	

2. Year of birth: ……….

3. Body weight (in kilograms): ……….

4. Height (in cm): ……….

5. Education:

a. Primary
b. Secondary
c. Vocational
d. Higher

6. Place of residence:

a. City up to 50k inhabitants
b. City up to 100k inhabitants
c. City up to 250k inhabitants
d. City over 250k inhabitants
e. Village

NCBR Narodowe Centrum Badań i Rozwoju

„Nowej generacji środki regulujące apetyt – użyteczne w opiece długoterminowej oraz kontroli masy ciała" Akronim RANG.e
Projekt finansowany przez Narodowe Centrum Badań i Rozwoju
Numer umowy: LIDER/48/0191/L-11/19/NCBR/2020

LIDER

COMPARISION

1. Please arrange the received samples from the least pleasant smelling to the most pleasant smelling:

Least pleasant scent	▼						Most pleasant scent

2. Please arrange the received samples from the least to the most appetite reducing:

Least appetite reducing	▼						Most appetite reducing

3. If you were to choose one scent to encounter on a daily basis, it would be: ………………………..
4. If you were to choose one scent to avoid encountering on a daily basis, it would be: ………………………..

NCBR Narodowe Centrum Badań i Rozwoju

„Nowej generacji środki regulujące apetyt – użyteczne w opiece długoterminowej oraz kontroli masy ciała" Akronim RANG.e
Projekt finansowany przez Narodowe Centrum Badań i Rozwoju
Numer umowy: LIDER/48/0191/L-11/19/NCBR/2020

LIDER

Supplementary materials: Instruction for participants

Natural Appetite Control: Food-Derived Aromas as Appetite Decreasing Agents - a Proof-of-Concept Study

Michaela Godyla-Jabłoński [1], Natalia Pachura [2], Marta Klemens [2], Julia Wolska [2] and Jacek Łyczko [2,*]

[1] Department of Human Nutrition, Wrocław University of Environmental and Life Sciences, ul. Chełmońskiego 37/41, 51-630 Wrocław, Poland; michaela.godyla@upwr.edu.pl

[2] Department of Food Chemistry and Biocatalysis, Wrocław University of Environmental and Life Sciences, ul. Chełmońskiego 37/41, 51-630, Wrocław, Poland; natalia.pachura@upwr.edu.pl; marta.klemens@upwr.edu.pl; 122508@student.upwr.edu.pl; jacek.lyczko@upwr.edu.pl

*Corresponding author. Email: jacek.lyczko@upwr.edu.pl

UNIWERSYTET
PRZYRODNICZY
WE WROCŁAWIU

LIDER PROJEKTU
Uniwersytet Przyrodniczy we Wrocławiu
Ul. C.K. Norwida 25
50-375 Wrocław

INSTRUCTIONS FOR PERFORMING THE TEST

1. All the documents to be completed are in the prepared plastic shirt.
2. Begin the study by completing the metric.
3. Proceed to the examination of individual samples. The received samples can be sniffed in any order.
4. After selecting a sample, find the sheet and compare the code on the sample with the code in the upper right corner of the sheet. Each sample is assigned an individual sheet.
5. Before testing the first sample, clean your nose by sniffing the enclosed tea bag. Do this activity before moving on to each subsequent sample.
6. Open the sample and sniff. Then, while constantly holding the sample to your nose, fill out the questionnaire.
7. When you have finished testing a particular sample, set it aside with the sheet.
8. Before testing the next sample, clean your nose by sniffing the enclosed tea bag. Do this activity before moving on to each subsequent sample.
9. After testing all samples (7), collect all samples together and fill out the "Comparison" sheet.

If you have any questions or concerns, ask the Members of the study team for assistance.

NCBR Narodowe Centrum Badań i Rozwoju

„Nowej generacji środki regulujące apetyt – użyteczne w opiece długoterminowej oraz kontroli masy ciała" Akronim RANG.e
Projekt finansowany przez Narodowe Centrum Badań i Rozwoju
Numer umowy: LIDER/48/0191/L-11/19/NCBR/2020

LIDER

Supplementary materials: HS-SPME-GC-MS results

Natural Appetite Control: Food-Derived Aromas as Appetite Decreasing Agents - a Proof-of-Concept Study

Michaela Godyla-Jabłoński [1], Natalia Pachura [2], Marta Klemens [2], Julia Wolska [2] and Jacek Łyczko [2,*]

[1] Department of Human Nutrition, Wrocław University of Environmental and Life Sciences, ul. Chełmońskiego 37/41, 51-630 Wrocław, Poland; michaela.godyla@upwr.edu.pl

[2] Department of Food Chemistry and Biocatalysis, Wrocław University of Environmental and Life Sciences, ul. Chełmońskiego 37/41, 51-630, Wrocław, Poland; natalia.pachura@upwr.edu.pl; marta.klemens@upwr.edu.pl; 122508@student.upwr.edu.pl; jacek.lyczko@upwr.edu.pl

*Corresponding author. Email: jacek.lyczko@upwr.edu.pl

Table S1 Volatiles profile of sample X034 based on HS-SPME-GC-MS analysis

LP.	Compound	RT [min]	LRI exp	LRI lit	A	B	C
					Area %	Area %	Area %
1	Pyrazine, 2,5-dimethyl- + Pyrazine, 2,6-dimethyl-	6.89	922	912	32.73	34.09	33.91
2	Propanoic acid, 2-methyl-, butyl ester	8.06	957	953	14.11	14.40	12.54
3	2,2-Dimethyl-1,3-dioxolane-4-carbaldehyde	8.71	977	962	45.97	43.71	44.31
4	Propane-1,1-diol diacetate	9.33	980	988	0.30	0.26	0.19
5	Myrcene	9.57	992	991	1.66	1.57	1.87
6	2,3,5-trimethyl-Pyrazine	9.99	1002	1002	0.16	0.15	0.20
7	Pyrazine, 2-ethyl-3-methyl-	10.59	1004	1001	0.36	0.39	0.34
8	p-Cymene	10.70	1025	1025	1.04	1.13	1.01
9	Butanoic acid, 2-methyl-, butyl ester	11.22	1041	1042	0.80	0.93	1.04
10	2(3H)-Furanone, 5-ethyldihydro-	13.16	1056	1060	0.39	0.47	0.62
11	Nonanal	16.21	1104	1107	0.11	0.11	0.11
12	IS	19.63	1294	1294	0.00	0.00	0.00
13	Butanoic acid, 2-butoxy-1-methyl-2-oxoethyl ester	21.48	1365	1357	1.47	1.72	2.37
14	Butanoic acid, 3-methyl-, octyl ester	23.42	1442	1441	0.13	0.15	0.21
15	Benzeneacetic acid, 3-methylbutyl ester	24.38	1495	1489	0.78	0.93	1.28

RT - retention time; LRI exp. - exerimentally obrained linear retention index; LRI lit. - litrature linear retention index; HS-SPME-GC-MS headspace solid-phase microextraction coupled with gas chromatography and mass spectrometry

This document was created by an application that isn't licensed to use novaPDF.
Purchase a license to generate PDF files without this notice.

Table S2 Volatiles profile of sample H090 based on HS-SPME-GC-MS analysis

LP.	Compound	RT [min]	LRI exp	LRI lit	A	B	C
					**Area %**	**Area %**	**Area %**
1	Butanoic acid, 1-methylethyl ester	4.45	849	835	0.33	0.35	0.34
2	Acetic acid, pentyl ester	6.02	896	884	0.04	0.04	0.04
3	β-Pinene	8.60	981	978	0.13	0.13	0.12
4	trans-meta-Mentha-2,8-diene	9.01	986	982	0.03	0.03	0.04
5	5-Hepten-2-one, 6-methyl-	9.05	989	986	5.24	5.37	5.41
6	Myrcene	9.32	993	991	36.24	36.57	36.32
7	Hexanoic acid, ethyl ester	9.50	1000	1003	0.04	0.04	0.04
8	p-Mentha-1(7),8-diene	9.78	1005	1004	3.15	3.19	3.24
9	Acetic acid, hexyl ester	9.95	1014	1012	0.12	0.12	0.13
10	p-Cymene	10.44	1026	1025	0.63	0.66	0.68
11	Limonene	10.71	1030	1030	50.83	50.35	50.19
12	Butanoic acid, 3-methylbutyl ester	11.42	1056	1057	0.15	0.15	0.16
13	Butanoic acid, 2-methylbutyl ester	11.49	1058	1056	0.05	0.06	0.06
14	Pentanoic acid, 4-oxo-, ethyl ester	11.58	1064	1065	0.03	0.03	0.02
15	cis-Linalool oxide	12.09	1074	1069	0.02	0.01	0.02
16	Hexanoic acid, 2-propenyl ester	12.26	1083	1081	1.54	1.52	1.64
17	trans-Linalool oxide	12.53	1090	1086	0.02	0.02	0.02
18	Linalool	12.95	1101	1101	1.22	1.19	1.33
19	cis-Limonene oxide	14.15	1136	1134	0.02	0.02	0.02
20	Camphor	14.34	1148	1149	0.09	0.09	0.12
21	Heptanoic acid, 2-propenyl ester	15.73	1181	1182	0.08	0.08	0.08
22	IS	19.66	1294	1294	0.00	0.00	0.00

RT - retention time; LRI exp. - exerimentally obrained linear retention index; LRI lit. - litrature linear retention index; HS-SPME-GC-MS headspace solid-phase microextraction coupled with gas chromatography and mass spectrometry

This document was created by an application that isn't licensed to use novaPDF.
Purchase a license to generate PDF files without this notice.

Table S3 Volatiles profile of sample F699 based on HS-SPME-GC-MS analysis

LP	Compound	RT [min]	LRI exp	LRI lit	A	B	C
					Area %	Area %	Area %
1	Tricyclene	7.37	933	923	0.40	0.35	0.41
2	α-Thujene	7.44	937	927	0.40	0.36	0.40
3	α-Pinene	7.67	943	933	0.75	0.65	0.72
4	Camphene	8.15	956	953	2.23	1.94	2.20
5	Sabinene	8.82	978	972	0.14	0.12	0.14
6	5-Hepten-2-one, 6-methyl-	9.07	989	986	21.86	19.13	20.60
7	Myrcene	9.28	993	991	2.55	2.16	2.46
8	α-Phellandrene	9.86	1007	1007	1.24	1.12	1.30
9	α-Terpinene	10.44	1018	1018	29.99	27.16	30.82
10	p-Cymene	10.62	1026	1025	1.50	1.31	1.51
11	Limonene	10.71	1032	1030	1.10	0.98	1.05
12	Eucalyptol	11.15	1034	1032	0.11	0.10	0.12
13	E-β-Ocimene	11.30	1049	1046	0.05	0.03	0.05
14	4-Nonanone	11.97	1073	1078	1.52	1.51	1.60
15	Linalool	12.96	1101	1101	0.17	0.18	0.18
16	trans-Chrysanthemol	14.70	1152	1152	0.07	0.09	0.09
17	Citronellal	14.79	1155	1152	0.08	0.08	0.08
18	Isogeranial	15.45	1166	1168	0.08	0.09	0.08
19	Borneol	15.56	1169	1173	0.08	0.10	0.09
20	Isoneral	15.76	1184	1174	0.01	0.01	0.01
21	Myrtenal	16.35	1197	1200	0.02	0.04	0.01
22	Verbenone	16.74	1214	1208	2.36	2.78	2.27
23	Neral	17.79	1244	1238	4.35	5.33	4.58
24	Carvone	18.01	1248	1246	20.79	24.19	20.85
25	Geraniol	18.21	1257	1255	0.27	0.36	0.29
26	Geranial	18.79	1274	1268	6.83	8.60	7.03
27	IS	19.65	1294	1294	0.00	0.00	0.00
28	α-Cubebene	20.20	1359	1349	0.10	0.06	0.06
29	Cyclosativene	21.69	1375	1367	0.01	0.01	0.01
30	α-Ylangene	22.02	1380	1371	0.16	0.18	0.17
31	α-Copaene	22.11	1384	1375	0.34	0.47	0.36
32	trans-Geranyl acetate	22.37	1388	1380	0.03	0.03	0.03
33	β-Bourbonene	22.47	1392	1382	0.04	0.04	0.04
34	trans-Caryophyllene	23.17	1430	1424	0.21	0.24	0.21
35	α-Humulene	23.89	1465	1454	0.04	0.05	0.05
36	δ-Cadinene	24.90	1527	1518	0.12	0.17	0.13

RT - retention time; LRI exp. - exerimentally obrained linear retention index; LRI lit. - litrature linear retention index; HS-SPME-GC-MS headspace solid-phase microextraction coupled with gas chromatography and mass spectrometry

This document was created by an application that isn't licensed to use novaPDF.
Purchase a license to generate PDF files without this notice.

Table S4 Volatiles profile of sample S005 based on HS-SPME-GC-MS analysis

LP.	Compound	RT [min]	LRI exp	LRI lit	A	B	C
					Area %	Area %	Area %
1	Pyrazine, 2,5-dimethyl- + Pyrazine, 2,6-dimethyl-	6.93	910	912	0.87	0.86	1.03
2	Thujene <alpha->	7.42	926	927	0.30	0.33	0.36
3	Pinene <alpha->	7.65	934	933	12.58	12.87	13.60
4	Citronellene <beta->	7.88	942	948	0.32	0.32	0.34
5	Camphene	8.13	950	953	1.37	1.33	1.43
6	Thuja-2,4(10)-diene	8.24	954	953	0.22	0.23	0.27
7	Sabinene	8.80	973	972	2.74	2.85	2.92
8	Pinene <beta->	8.97	979	978	11.38	11.83	12.22
9	Menth-3-ene <para->	9.17	985	986	0.37	0.35	0.37
10	Myrcene	9.26	988	991	3.43	3.46	3.10
11	3-Octanol	9.45	995	999	0.95	0.94	0.97
12	Pyrazine, 2-ethyl-3-methyl-	9.60	999	1001	0.26	0.27	0.29
13	Mentha-1(7),8-diene <p->	9.77	1005	1004	0.20	0.19	0.20
14	Carene <delta-3->	9.94	1010	1009	0.09	0.10	0.10
15	Terpinene <alpha->	10.20	1018	1018	0.10	0.09	0.10
16	Cymene <para->	10.42	1025	1025	1.11	1.10	1.06
17	Limonene	10.60	1029	1030	23.73	23.62	22.37
18	Eucalyptol	10.70	1032	1032	0.55	0.55	0.54
19	Ocimene <(E)-, beta->	11.14	1045	1046	0.08	0.08	0.08
20	Terpinene <gamma->	11.57	1058	1058	0.24	0.24	0.23
21	Terpinolene	12.52	1086	1086	0.11	0.10	0.09
22	Isopulegol	14.69	1148	1149	0.23	0.22	0.22
23	Menthone	14.94	1155	1158	17.29	17.24	16.74
24	Isomenthone	15.25	1164	1166	6.72	6.63	6.68
25	Neomenthol	15.43	1169	1170	1.91	1.87	1.92
26	Menthol	15.70	1179	1184	9.28	8.87	9.27
27	Menthol <iso->	16.11	1188	1185	0.23	0.23	0.22
28	1-Dodecene	16.22	1191	1191	0.07	0.07	0.07
29	Terpineol <alpha->	16.33	1195	1195	0.10	0.09	0.11
30	Pulegone	17.83	1238	1241	0.22	0.21	0.22
31	Piperitone	18.37	1259	1267	0.15	0.16	0.16
32	Menthyl acetate	19.59	1289	1290	2.05	1.99	2.06
33	IS	19.64	1294	1294	0.00	0.00	0.00
34	Elemene <delta->	21.00	1339	1335	0.06	0.06	0.03
35	Bourbonene <beta->	22.36	1389	1382	0.32	0.30	0.29
36	Longifolene	22.96	1416	1412	0.02	0.01	0.01
37	Caryophyllene <(E)->	23.16	1426	1424	0.28	0.26	0.26
38	Copaene <beta->	23.35	1437	1433	0.04	0.04	0.04
39	Isogermacrene D	23.79	1451	1447	0.05	0.04	0.04

RT - retention time; LRI exp. - exerimentally obrained linear retention index; LRI lit. - litrature linear retention index; HS-SPME-GC-MS headspace solid-phase microextraction coupled with gas chromatography and mass spectrometry

This document was created by an application that isn’t licensed to use novaPDF.
Purchase a license to generate PDF files without this notice.

Table S5 Volatiles profile of sample R789 based on HS-SPME-GC-MS analysis

LP.	Compound	RT [min]	LRI exp	LRI lit	A	B	C
					Area %	Area %	Area %
1	Furfur-3-al	5.06	820	816	11.23	11.64	11.13
2	Thujene <alpha->	7.37	925	927	0.11	0.17	0.11
3	Pinene <alpha->	7.71	936	933	86.92	86.33	87.10
4	Camphene	8.15	951	953	0.73	0.80	0.75
5	Pinene <beta->	8.99	980	978	0.32	0.31	0.31
6	Cymene <para->	10.45	1025	1025	0.24	0.26	0.22
7	Pinene oxide <alpha->	13.01	1100	1101	0.09	0.09	0.08
8	Mentha-2,8-dien-1-ol <trans-, p->	13.35	1117	1122	0.07	0.07	0.06
9	Campholenic aldehyde <alpha->	13.94	1127	1126	0.21	0.25	0.19
10	Pinocarveol <trans->	14.47	1142	1141	0.02	0.02	0.02
11	Verbenol <trans->	14.62	1146	1145	0.03	0.03	0.03
12	Pinocamphone <cis->	15.15	1164	1176	0.03	0.03	0.03
13	IS	19.67	1294	1294	0.00	0.00	0.00

RT - retention time; LRI exp. - exerimentally obrained linear retention index; LRI lit. - litrature linear retention index; HS-SPME-GC-MS headspace solid-phase microextraction coupled with gas chromatography and mass spectrometry

This document was created by an application that isn't licensed to use novaPDF.
Purchase a license to generate PDF files without this notice.

Table S6 Volatiles profile of sample D418 based on HS-SPME-GC-MS analysis

**LP.**	**Compound**	**RT [min]**	**LRI exp**	**LRI lit**	**A**	**B**	**C**
					**Area %**	**Area %**	**Area %**
1	2-Heptanone	6.34	886	898	66.92	68.66	66.96
2	Nonane	6.68	901	900	0.14	0.16	0.12
3	Pinene <alpha->	7.66	935	933	0.4	0.39	0.37
4	Camphene	8.14	951	953	0.07	0.07	0.06
5	Hept-5-en-2-one <6-methyl->	9.06	982	986	27.88	26.8	27.95
6	Cymene <para->	10.43	1025	1025	0.18	0.17	0.18
7	Limonene	10.61	1030	1030	0.11	0.1	0.11
8	Eucalyptol	10.72	1033	1032	0.71	0.64	0.67
9	Nonanal	13.11	1103	1107	0.07	0.06	0.08
10	Camphor	14.67	1148	1149	0.06	0.06	0.07
11	Decanal	16.68	1205	Q208	3.46	2.89	3.44
12	IS	19.66	1294	1294	0	0	0

RT - retention time; LRI exp. - exerimentally obrained linear retention index; LRI lit. - litrature linear retention index; HS-SPME-GC-MS headspace solid-phase microextraction coupled with gas chromatography and mass spectrometry

This document was created by an application that isn’t licensed to use novaPDF.
Purchase a license to generate PDF files without this notice.

Supplementary materials: statistical assumptions

Natural Appetite Control: Food-Derived Aromas as Appetite Decreasing Agents - a Proof-of-Concept Study

Michaela Godyla-Jabłoński [1], Natalia Pachura [2], Marta Klemens [2], Julia Wolska [2] and Jacek Łyczko [2,*]

[1] Department of Human Nutrition, Wrocław University of Environmental and Life Sciences, ul. Chełmońskiego 37/41, 51-630 Wrocław, Poland; michaela.godyla@upwr.edu.pl

[2] Department of Food Chemistry and Biocatalysis, Wrocław University of Environmental and Life Sciences, ul. Chełmońskiego 37/41, 51-630, Wrocław, Poland; natalia.pachura@upwr.edu.pl; marta.klemens@upwr.edu.pl; 122508@student.upwr.edu.pl; jacek.lyczko@upwr.edu.pl

*Corresponding author. Email: jacek.lyczko@upwr.edu.pl

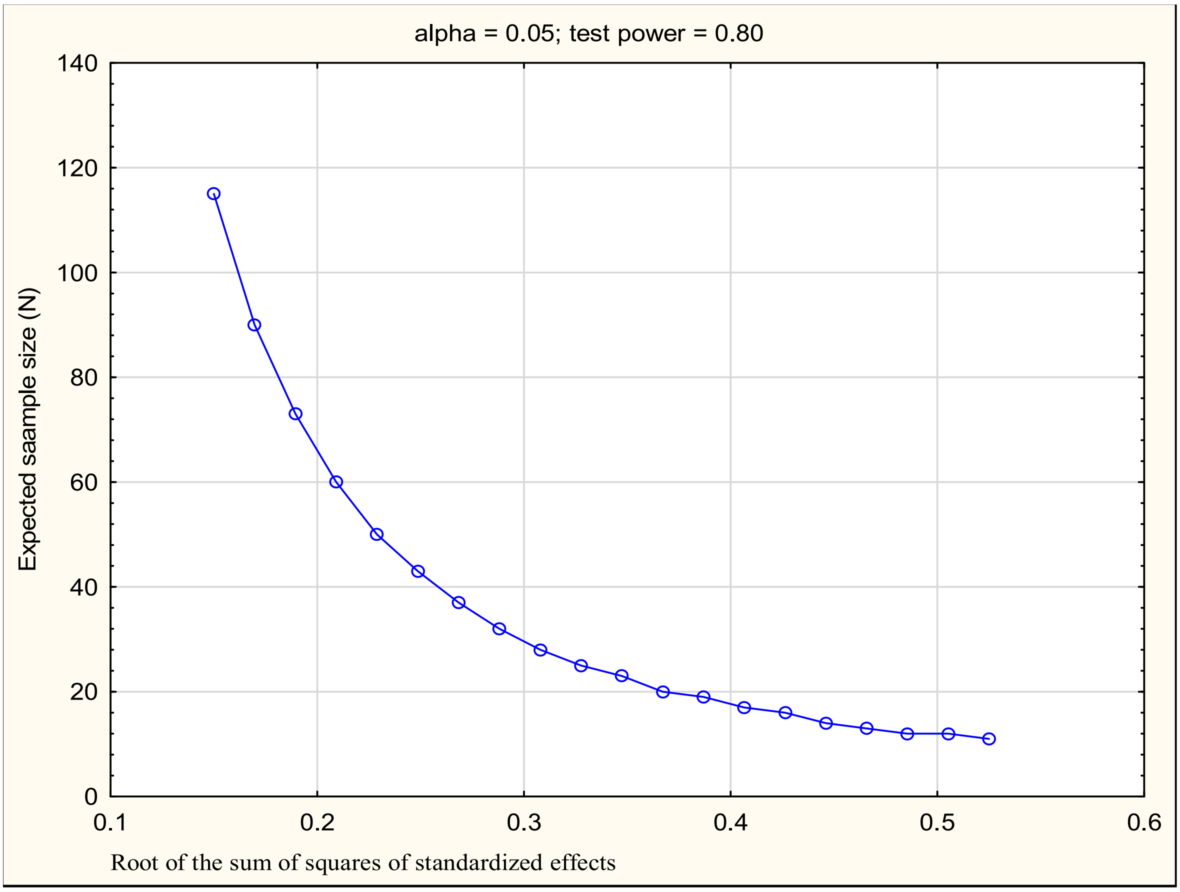

Figure S1 Sumple size approximation

This document was created by an application that isn't licensed to use novaPDF.
Purchase a license to generate PDF files without this notice.

Table S7 Data set and calculation of Cronbach's coefficient alpha

	**Q1**	**Q2**	**Q2***	**Q3**	**Q4**	**Q5**	**SUM**
1	4	1	2	4	3	5	19
2	4	3	0	0	3	4	14
3	2	1	1	1	1	1	7
4	3	1	0	1	1	3	9
5	2	2	1	1	1	3	10
6	5	2	2	4	3	3	19
7	3	2	2	1	1	3	12
8	4	3	0	1	2	4	14
9	4	3	0	4	2	4	17
10	2	1	1	1	1	1	7
variance	1.1222222	0.7666667	0.76666667	2.4	0.844444444	1.65555556	20.8444444
SUM of variance	7.555555556						
Cronbach's alpha	0.796908316						

This document was created by an application that isn’t licensed to use novaPDF.
Purchase a license to generate PDF files without this notice.

Table S8 Matrix of non-standarized and standarized data for heatmap creation

Raw data						
	Q1	Q2a	Q2b	Q3	Q4	Q5
D418	2.00	5.00	0.00	1.00	-5.00	4.00
F699	4.00	5.00	0.00	2.00	5.00	2.00
H090	4.00	5.00	0.00	4.00	-5.00	4.00
L368	3.00	-5.00	1.00	1.00	-5.00	3.00
R789	2.00	5.00	0.00	1.00	-5.00	2.00
S005	4.00	5.00	0.00	1.00	-5.00	2.00
X034	5.00	5.00	0.00	4.00	5.00	1.00
Standarized data						
	Q1	Q2	Q2*	Q3	Q4	Q5
D418	-0.87	0.33	0.06	-0.74	-0.67	0.42
F699	0.90	0.67	-0.88	0.39	0.83	-0.23
H090	0.22	0.54	-0.69	0.31	0.08	1.06
L368	-0.69	-2.14	1.45	-1.10	-1.13	-1.45
R789	-1.05	-0.35	1.32	-0.58	-0.90	0.30
S005	-0.19	0.46	-0.38	-0.20	0.13	1.06
X034	1.67	0.50	-0.88	1.90	1.66	-1.16

This document was created by an application that isn't licensed to use novaPDF.
Purchase a license to generate PDF files without this notice.

Supplementary materials: ANOVA

Natural Appetite Control: Food-Derived Aromas as Appetite Decreasing Agents - a Proof-of-Concept Study

Michaela Godyla-Jabłoński [a], Natalia Pachura [b], Marta Klemens [b], Julia Wolska [b] and Jacek Łyczko [b,*]

[a] Department of Human Nutrition, Wrocław University of Environmental and Life Sciences, 51-630 Wrocław, Poland; michaela.godyla@upwr.edu.pl

[b] Department of Food Chemistry and Biocatalysis, Wrocław University of Environmental and Life Sciences, 50-375, Wrocław, Poland; natalia.pachura@upwr.edu.pl; marta.klemens@upwr.edu.pl; 122508@student.upwr.edu.pl; jacek.lyczko@upwr.edu.pl

*Corresponding author. Email: jacek.lyczko@upwr.edu.pl

Table S9 The influence of study participants sex, age, time form last meal and declared level of hunger on the evaluation of -ARAs samples potential for appetite reduction

Sex	Age [years]	Last meal [h]	Hunger level	Potential for appetite reduction points	ANOVA*
Women	44	1-2	hard to say	14.17	****
Men	47	2-3	full	15.83	****
Women	30	3-4	hungry	16.67	****
Men	24	>4	hungry	16.67	****
Men	22	2-3	hard to say	16.67	****
Women	23	>4	full	17.50	****
Men	30	1-2	full	18.33	****
Women	30	2-3	full	18.33	****
Women	31	2-3	hungry	18.33	****
Women	26	2-3	full	18.33	****
Women	26	>4	hungry	18.33	****
Women	44	2-3	full	19.17	****
Women	37	3-4	full	19.17	****
Women	42	2-3	hard to say	19.17	****
Women	43	>4	hard to say	20.00	****
Women	38	2-3	full	20.00	****
Women	41	>4	very hungry	20.00	****
Women	37	1-2	full	20.83	****
Women	25	>4	hungry	20.83	****
Women	50	<1	full	20.83	****
Women	50	>4	full	20.83	****
Men	50	1-2	hard to say	20.83	****
Men	31	2-3	hard to say	20.83	****
Women	43	1-2	full	20.83	****
Women	49	2-3	hard to say	21.67	****
Women	36	<1	full	21.67	****
Men	19	>4	hungry	21.67	****
Women	31	1-2	full	21.67	****
Women	50	3-4	full	21.67	****
Women	26	1-2	hard to say	21.67	****
Women	23	1-2	hungry	21.67	****
Women	36	2-3	hard to say	21.67	****
Women	36	1-2	full	21.67	****
Men	29	>4	hard to say	21.67	****
Women	47	1-2	hard to say	21.67	****
Women	23	1-2	hard to say	21.67	****
Women	58	1-2	full	21.67	****
Women	32	1-2	full	22.50	****
Women	51	2-3	hard to say	22.50	****
Women	22	1-2	full	22.50	****
Women	40	3-4	hard to say	22.50	****
Women	31	3-4	hard to say	23.33	****
Men	19	>4	hard to say	24.17	****

*Significant at $p < 0.05$

This document was created by an application that isn't licensed to use novaPDF.
Purchase a license to generate PDF files without this notice.

Table S10 The influence of study participants sex, age, time form last meal and declared level of hunger on the evaluation of -ARAs samples pleasure of aroma

Sex	Age [years]	Last meal [h]	Hunger level	Pleasure of aroma points	ANOVA*
Women	26	>4	hungry	16.67	****
Women	31	2-3	hungry	17.50	****
Women	36	1-2	full	18.33	****
Women	25	>4	hungry	18.33	****
Men	24	>4	hungry	18.33	****
Men	22	2-3	hard to say	18.33	****
Women	50	3-4	full	19.17	****
Women	58	1-2	full	19.17	****
Men	50	1-2	hard to say	19.17	****
Women	38	2-3	full	20.00	****
Men	30	1-2	full	20.00	****
Men	19	>4	hungry	20.00	****
Women	26	2-3	full	20.00	****
Women	36	2-3	hard to say	20.00	****
Women	22	1-2	full	20.00	****
Women	32	1-2	full	20.00	****
Women	30	2-3	full	20.00	****
Women	43	>4	hard to say	20.00	****
Men	47	2-3	full	20.00	****
Women	44	2-3	full	20.00	****
Women	43	1-2	full	20.42	****
Women	49	2-3	hard to say	20.42	****
Women	50	>4	full	20.83	****
Women	31	3-4	hard to say	20.83	****
Women	30	3-4	hungry	20.83	****
Women	37	1-2	full	20.83	****
Women	23	1-2	hungry	20.83	****
Women	40	3-4	hard to say	21.67	****
Men	19	>4	hard to say	21.67	****
Women	44	1-2	hard to say	21.67	****
Women	31	1-2	full	21.67	****
Women	51	2-3	hard to say	21.67	****
Women	23	>4	full	21.67	****
Women	47	1-2	hard to say	22.50	****
Women	26	1-2	hard to say	22.50	****
Women	41	>4	very hungry	22.50	****
Women	50	<1	full	22.50	****
Women	42	2-3	hard to say	22.50	****
Women	37	3-4	full	22.50	****
Men	29	>4	hard to say	22.50	****
Women	36	<1	full	22.50	****
Men	31	2-3	hard to say	24.17	****
Women	23	1-2	hard to say	25.83	****

*Significant at $p < 0.05$

This document was created by an application that isn't licensed to use novaPDF.
Purchase a license to generate PDF files without this notice.
